# A Case of Bilateral Spontaneous Chylothorax with Respiratory Syncytial Virus Bronchiolitis

**DOI:** 10.1155/2019/2853632

**Published:** 2019-02-06

**Authors:** Mario Briceno-Medina, Michael Perez, Jie Zhang, Ronak Naik, Samir Shah, Dai Kimura

**Affiliations:** ^1^Division of Cardiology, Le Bonheur Children's Hospital, University of Tennessee Health Science Center, Memphis, TN, USA; ^2^Division of Pediatric Cardiology, Ann & Robert H. Lurie Children's Hospital of Chicago, Northwestern University, Chicago, IL, USA; ^3^Department of Pathology, Le Bonheur Children's Hospital, University of Tennessee Health Science Center, Memphis, TN, USA; ^4^Division of Critical Care Medicine, Le Bonheur Children's Hospital, University of Tennessee Health Science Center, Memphis, TN, USA

## Abstract

A case of bilateral spontaneous chylothorax with respiratory syncytial virus (RSV) bronchiolitis has never been reported. We report the case of a 7-month-old boy born at 33 weeks gestation with a history of Down syndrome, atrial septal defect, pulmonary hypertension, and chronic lung disease, hospitalized due to RSV bronchiolitis who developed bilateral spontaneous chylothorax with exacerbation of pulmonary hypertension (PH). The patient died after 9 weeks of mechanical ventilation and treatment for PH. The autopsy showed acute infectious signs, a chronic interstitial lung disease with pulmonary hypertensive changes and subpleural cysts with no evidence of congenital lymphangiectasia. The cause of chylothorax in this child could be multifactorial. However, worsening pulmonary hypertension with RSV infection might have partially contributed to the development of chylothorax through elevated superior venous cava pressure. Thoracentesis should be considered for patients with Down syndrome and PH associated with congenital heart disease who develop persistent pleural effusion during RSV bronchiolitis to rule out chylothorax.

## 1. Introduction

Chylothorax, the accumulation of chylous pleural effusion, is very rare after neonatal period [1]. It can cause respiratory failure, malnutrition, and immunodeficiency if not treated. The cause of chylothorax includes lymphatic abnormality (pulmonary lymphangiomas and lymphangiectasia), trauma to the thoracic duct, venous thrombus in the superior vena cava (SVC) or subclavian vein, tumors such as lymphoma, and granulomatous infections such as tuberculosis, histoplasmosis, and sarcoidosis [1]. Chylothorax caused by other infectious diseases is extremely rare.

## 2. Case Description

The patient is a 7-month-old twin boy who presented to our institution's emergency department with increased work of breathing and desaturations (70 s). He was born at 33 weeks gestational age with Down syndrome, developed chronic lung disease (CLD) of prematurity, and was also found to have a moderate size secundum atrial septal defect (ASD) as a newborn. Prior to the current illness, he had been in the hospital multiple times for failure to thrive and respiratory distress, requiring mechanical ventilation with high amount of supplemental O_2_ and inhaled nitric oxide (iNO) as he developed pulmonary hypertension (PH). Echocardiography showed progressive enlargement and hypertrophy of his right ventricle and at times bidirectional shunting across his ASD. A diagnostic cardiac catheterization as a preoperative evaluation was performed, which showed elevated pulmonary vascular resistance indexed (PVRi) at baseline (8.8 WU·m^2^), which decreased with inhaled oxygen alone and iNO (3.8 WU·m^2^). Additional catheterization data at baseline condition showed a right atrial mean pressure of 6 mmHg, right ventricular end diastolic pressure of 6 mmHg, and pulmonary artery pressure 51/19 mmHg with mean 32 mmHg. The patient was started on home O_2_ therapy with nasal cannula. The current hospitalization occurred prior to a planned fenestrated patch repair of his ASD.

**Figure 1:**
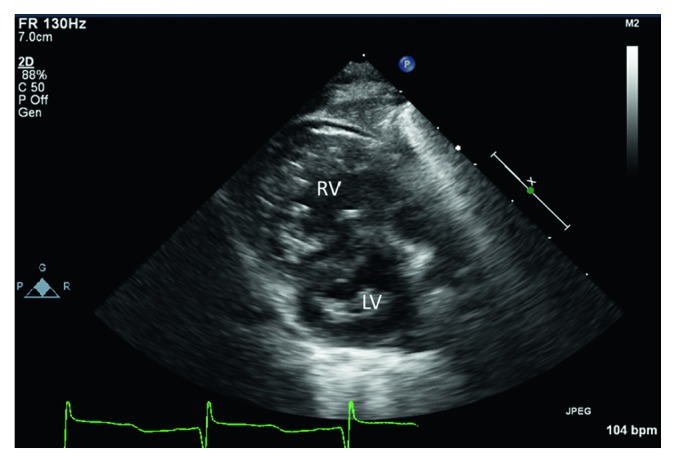
Parasternal short-axis view showing a flattening of the intraventricular septum during systole. Enlargement and hypertrophy of the right ventricle are also shown. RV, right ventricle; LV, left ventricle.

**Figure 2:**
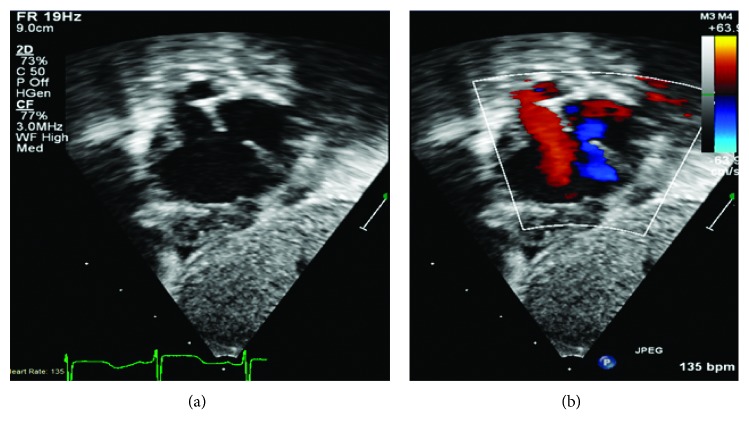
Color compared from the subcostal coronal view that shows moderate size secundum ASD with bidirectional shunting supporting findings of elevated RV pressure.

**Figure 3:**
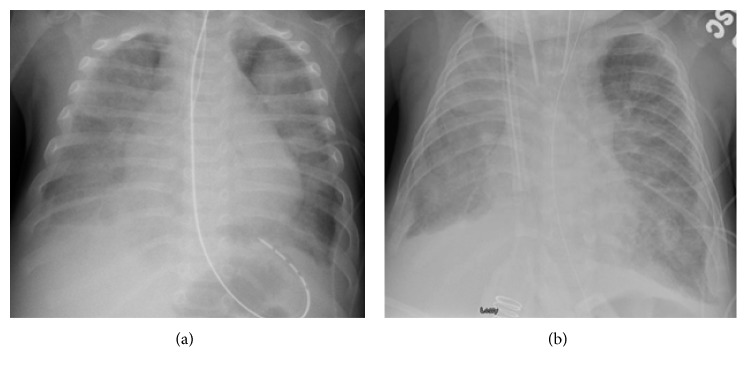
(a) Chest X-ray on admission showing bilateral pulmonary edema with pleural effusions. (b) Pleural effusion unresponsive to therapy with diuretics (hospital day #6).

He was initially admitted to the general ward and soon transferred to the pediatric ICU for severe hypoxemic respiratory failure requiring mechanical ventilation. Respiratory syncytial virus (RSV) infection was diagnosed with the positive antigen test. He continued to have paroxysmal severe hypoxic events compatible with PH crisis. He was treated with sedation and neuromuscular paralysis, increased FiO_2_, optimization of O_2_ carrying capacity with packed red blood cells transfusions, and iNO. Milrinone infusion was added as the right ventricular function was depressed on echocardiogram (TAPSE 6 mm, *Z*-score −4), which demonstrated evidence of systemic to suprasystemic right ventricular pressure and bidirectional shunting across the ASD (Figures 1 and 2). No other cardiovascular intravenous drips were given during the ICU stay. Sildenafil was initiated enterally and escalated to maximal dose (2 mg/kg/day) without hemodynamic compromise. He was on diuretic therapy (bumetanide infusion up to 10 mcg/kg/hr) as chest X-ray demonstrated evidence of bilateral interstitial edema with bilateral pleural effusions on admission (Figure 3) and confirmed by chest ultrasound. Bilateral chest tubes were placed after failure of diuretic therapy to reduce effusions on hospital day #6. The drained fluid was milky in appearance bilaterally, with a white blood cell of 1,004/mm^3^ with lymphocyte predominance (88%) and elevated triglycerides (1008 mg/dl), and hence a diagnosis of chylothorax was made. Low IgG level (249 mg/dl) and hypoalbuminemia (2.5 g/dl) were noted at the time of pleural effusion drainage. Intravenous immunoglobulin and 25% albumin solution were administered. His feeding formula was changed to medium-chain triglyceride formula. The milky drainage became serous; however, the volume of chest tube drainage remained unchanged. Enteral feeding was discontinued and total parenteral nutrition was initiated, which decreased the volume of pleural effluent but small to moderate amount of pleural effusion was intermittently observed by chest X-ray for over sixty three days until the patient's death. Venous Doppler ultrasound of the upper extremities and the neck was performed on hospital day #7 and 4 weeks later, and compression, thrombosis or obstruction of the superior vena cava, and upper extremity were ruled out. A central venous catheter was placed in the right jugular vein soon after admission and was removed on hospital day #7 and replaced by a peripherally inserted central line. The patient required chest tubes for drainage until hospital day #22. Since then, intermittently small to moderate pleural effusion was observed by chest X-ray, but chest tubes were not placed.

He continued to be critically ill with persistent hypoxemic respiratory failure without improvement in PH with several PH crisis episodes. Therapy with an endothelin (ET) receptor antagonist (Bosentan) was added. The hospital course was complicated by bacterial tracheitis from *Pseudomonas* and *E. coli*. The patient remained on mechanical ventilator support for 6 weeks due to failed weaning of ventilator support from hypoxemia despite high levels of supplementary FiO_2_ and iNO. Cardiac catheterization performed 6 weeks after admission showed PVRi of 7 WU m^2^ on 100% FiO_2_ and 20 ppm of iNO under general anesthesia, pulmonary venous desaturation, and bidirectional shunting through ASD. Additionally, interval increases in right atrial pressure (mean 13 mmHg), right ventricular end diastolic pressure (12 mmHg), and pulmonary artery pressure (52/24 mean 36 mmHg) were noted. Given his severe and irreversible lung injury from mechanical ventilation in addition to baseline chronic lung disease, he was deemed not a candidate for lung transplant. Considering that the patient had Eisenmenger physiology due to severe PH and poor prognosis, the palliative care team was also consulted. Weaning from the mechanical ventilator was tried multiple times, but failed. At 9 weeks of his ICU hospitalization, he developed severe hypoxemia unresponsive to medical therapy that ultimately caused his death.

**Figure 4:**
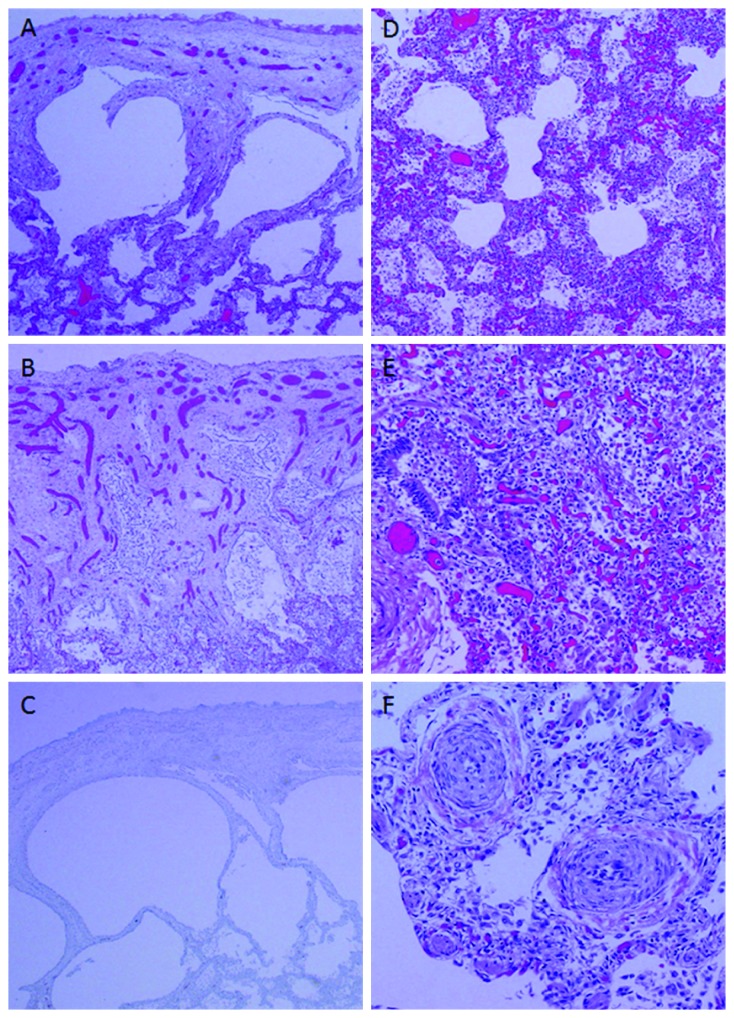
(A–C) Subpleural cysts lined by pneumocytes are seen in both lungs which contain macrophages, sloughed pneumocytes, and neutrophils ((A) H&E ×40 and (B) H&E ×20). (C) Immunohistochemistry with anti-D2-40 antibody, a marker for lymphatic endothelium, was negative. (D) Lung parenchyma demonstrates chronic interstitial lung disease with alveolar septal thickening, capillary disorganizing, and hemosiderosis (H&E ×40). (E) Acute bronchopneumonia is present (H&E ×40). (F) Pulmonary hypertensive changes are present with variable severities (H&E ×100).

An autopsy showed bilateral small straw-colored pleural effusions (right 17 ml and left 10 ml), and the lung parenchyma was red-brown, poorly aerated, and diffusely congested with focal consolidation. The heart had an ASD (0.8 × 1.2 cm) with right ventricular hypertrophy secondary to PH. Microscopically, both lungs showed subpleural cysts lined by pneumocytes and containing macrophages, sloughed pneumocytes, and neutrophils. Acute multifocal bronchopneumonia was present with neutrophils in the bronchioles and alveoli. Chronic interstitial lung disease is diffusely present with alveolar septal thickening, capillary disorganization, and hemosiderosis. Small pulmonary arterial branches demonstrate moderate to marked medial smooth muscle hypertrophy with lumen narrowing, while large pulmonary arteries were normal with minimal changes. No lymphatic dilatation was observed on H&E or D2-40 immunostained slides; therefore, lymphangiectasia was ruled out (Figure 4). From the autopsy results, hypoxia due to progressive PH was considered as a cause of death.

## 3. Discussion

Chylothorax with granulomatous infection has been reported; however, there are no case reports of bilateral spontaneous chylothorax due to acute RSV bronchiolitis or bronchiolitis due to other viral infection [1]. The suspected mechanism of chylothorax in this child is multifactorial. However, worsening of PH with RSV bronchiolitis might have partially contributed to the development of chylothorax via elevated SVC pressure. PVRi measured by catheterization in this patient was not significantly different from the baseline, but the procedure was conducted at 6 weeks of admission after improvement in hypoxia. We suspect PVRi was even higher when the patient had severe respiratory failure with bilateral chylothorax soon after admission. Elevated SVC pressure is one of the etiologies of chylothorax resulting from the thoracic duct flow obstruction [2]. Another possible factor is an obstruction of SVC by a central venous catheter in the right jugular vein even though Doppler ultrasound exams did not show signs of SVC thrombus and chest tube drainage continued until hospital day #22. We conducted Doppler ultrasound only twice and cannot completely rule out SVC thrombus before or between Doppler ultrasound exams. Many other factors possibly contributed to the development of chylothorax, including PH from congenital heart disease (CHD), chronic lung disease (CLD) of prematurity, and Down syndrome. Thoracentesis should be considered in patients with those risk factors for PH who develop persistent pleural effusion during RSV bronchiolitis to rule out chylothorax. Therapeutic drainage with chest tube placement may be necessary in patients with respiratory failure due to pleural effusions.

Transient or worsening PH in children with moderate to severe acute bronchiolitis has been reported and associated with a longer length of stay in the hospital [3–5]. The presence of PH in children with RSV infection was particularly associated with severity of the illness, with a mortality of 73% for children with CHD and PH [6]. Cardiac surgery for children with CHD performed during the symptomatic period of RSV infection is associated with a high risk of postoperative complications, especially postoperative PH, and in a rare case, chylous effusion [7]. In the report, three patients with Down syndrome, CHD and PH died from RSV infection and could not receive cardiac surgery [7]. The pathophysiology of the development of PH during RSV bronchiolitis is unknown. In RSV bronchiolitis, hyperinflation and atelectasis secondary to lower airway obstruction and eventual hypoxia and hypercapnia can also cause increased pulmonary vascular resistance [8]. Therefore, multiple factors likely contribute to the development of PH in RSV bronchiolitis.

Patients with Down syndrome and CHD have a higher risk of developing PH quicker and with more damage to the pulmonary vascular bed than those without Down syndrome [9]. Persistently elevated serum ET-1 levels were observed in patients with Down syndrome after cardiopulmonary bypass for cardiac operations [10]. The imbalance between the prostacyclin and thromboxane and decreased arginine and NO production are other possible mechanisms of PH in Down syndrome [11]. Patients with Down syndrome also have a higher risk of developing chylothorax, most commonly congenitally [12, 13]. Furthermore, infants with Down syndrome were more likely to develop chylothorax after operation for CHD [14]. The etiology of chylothorax in patients with Down syndrome is not well understood, but it is likely associated with anomalous lymph drainage associated with aneuploidy syndromes (trisomy, Turner's syndrome, and Noonan's syndrome) [15]. In this patient, an autopsy showed subpleural cysts; however, there was no sign of congenital lymphangiectasis.

## 4. Conclusion

For patients with Down syndrome and PH associated with CHD who develop persistent pleural effusion during RSV bronchiolitis, thoracentesis should be considered to rule out chylothorax.
